# Beyond the Neonate: A Delayed Presentation of Congenital Diaphragmatic Hernia in a 17-Year-Old

**DOI:** 10.1155/2024/7518183

**Published:** 2024-05-15

**Authors:** Vivienne Vinton, Molly Posa, Maria N. Kelly, Janice A. Taylor, Jaclyn Otero

**Affiliations:** ^1^University of Florida College of Medicine, Shands Hospital, 1600 SW Archer Road, P.O. Box 100209, Gainesville, FL 32608, USA; ^2^UF Department of Pediatrics, 133 SW 130^th^ Way, Suite C, Newberry, FL 32669, USA; ^3^Division of General Academic Pediatrics, 1329 SW 16th Street, Suite 2170, Gainesville, FL 32608, USA; ^4^Pediatric Surgery, UF Department of Surgery, 1600 SW Archer Road, P.O. Box 100119, Gainesville, FL 32610, USA; ^5^UF Department of Pediatrics, 4740 NW 39th PI B, Gainesville, FL 32606, USA

## Abstract

Congenital diaphragmatic hernia (CDH) is a rare anomaly resulting from incomplete closure of pleuroperitoneal canals during fetal development, often presenting with acute respiratory distress in neonates. This case report highlights a 17-year-old female with recurrent episodes of acute left upper quadrant (LUQ) pain and no history of trauma or dietary change. A computerized tomography (CT) scan taken during her second presentation to the emergency department led to a diagnosis of left-sided CDH. She later had a successful laparoscopic diaphragmatic repair surgery and has remained symptom-free for over a year. Late-presenting CDH indicates a rare subset of cases diagnosed after one month of age. Late presentations comprise 5–25% of cases and become increasingly rare with age. Unlike neonatal CDH, which is associated with several comorbidities, late presentations often manifest as a standalone anomaly. When the correct diagnosis is made, uncomplicated surgical repair yields excellent long-term outcomes. However, delayed and incorrect diagnoses can result in serious morbidity. Late-presenting CDH has diverse clinical presentations and can elude diagnostic imaging. As a result, there is a need for heightened clinical suspicion. This report aims to enhance awareness of late-presenting CDH and explore challenges to prompt, accurate diagnosis. Ultimately, this study implores clinicians to consider this condition in patients with unexplained respiratory or gastrointestinal symptoms.

## 1. Introduction/Background

Congenital diaphragmatic hernia (CDH) is an abnormality associated with incomplete closure of the pleuroperitoneal canals during fetal development. Openings in the pleuroperitoneal canals allow abdominal viscera to breach the chest cavity. These openings can be graded from A to D, with a grade A defect affecting up to 25% of the diaphragm and a grade D defect indicating diaphragmatic aplasia. Indeed, the grade of the defect correlates with the severity of symptoms [1]. Unfortunately, the most common presentation of CDH is an infant with a higher grade diaphragmatic defect (grades B, C) with acute respiratory distress within the first hours to days of life [1, 2]. In this presentation, CDH is associated with high infant mortality, affecting approximately 0.03% of newborns [2]. However, there is a subset of patients with a milder form who are diagnosed more than a month after birth, called late-presenting CDH [3–5]. Late-presenting CDH is estimated to account for 5–25% of all CDH diagnoses and becomes increasingly rare with age [3, 4, 6].

Neonatal CDH frequently presents with a higher grade defect, associated with pulmonary hypoplasia, pulmonary hypertension, and cardiac issues. On the other hand, late presentations often manifest as a lower grade defect and a standalone anomaly [2]. Accurate diagnosis followed by prompt surgical repair is associated with favorable outcomes; however, misdiagnosis can result in serious morbidity [3–5]. Our case presentation highlights a 17-year-old female with left upper quadrant (LUQ) pain found to have a diaphragmatic hernia requiring surgical repair.

## 2. Case Presentation

A 17-year-old female with a history of anxiety presented to the emergency department (ED) with an 18-hour history of intense LUQ abdominal pain that worsened with movement. She had no recent abdominal trauma or dietary changes but had previously experienced similar episodes of pain twice, with the most recent episode two years ago. She had normal vital signs, urinalysis, abdominal ultrasound, and a negative urine pregnancy test. Her pain improved in the ED, and she was discharged home.

**Figure 1:**
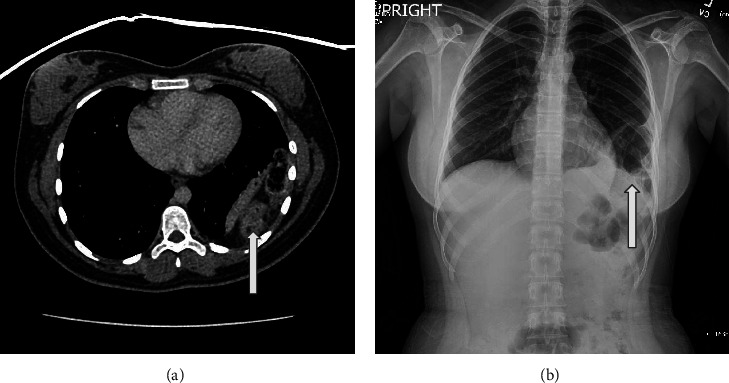
Imaging studies depicting bowel in the left lung field with defects in the left hemidiaphragm (arrows). (a) Chest CT. (b) Chest radiograph.

One month later, the patient returned to the ED with similar LUQ abdominal pain. She rated the pain as a 7/10 and noted it limited ambulation. She also reported chest tightness when lying flat, nausea, and one episode of nonbloody, nonbilious emesis. Her vital signs, complete blood count, urinalysis, complete metabolic panel, and lipase were within normal limits. An abdominal computerized tomography (CT) scan revealed a left hemidiaphragm defect with colon above the left diaphragm (Figure 1). These findings were absent on her recent abdominal ultrasound and a chest radiograph obtained 14 years prior. The patient was diagnosed with congenital diaphragmatic hernia (CDH), admitted overnight for pain control, and discharged home the following morning with surgical repair scheduled in one month.

However, two days later, the patient returned to the ED when severe LUQ abdominal pain caused a syncopal episode. She was admitted for three days for pain management. She had improvement with a combination of acetaminophen, ibuprofen, gabapentin, and oxycodone. Once discharged, she minimized physical exertion and adhered to a pain control and bowel regimen. One month later, she had successful laparoscopic diaphragmatic repair and has remained symptom-free for over a year.

## 3. Discussion

LUQ abdominal pain lends to a differential diagnosis of disorders involving the spleen, pancreas, neighboring muscles and bones, and raises suspicion for constipation. Emergent diagnoses include splenic enlargement, rupture, or infarction and pancreatitis. With a normal temperature, spleen size, and peripheral blood smear, splenic conditions are less likely [7]. When a patient presents with significant LUQ pain without obvious splenic etiology, pancreatitis, preceding musculoskeletal injury, or signs of constipation, the diagnosis of left-sided late-presenting congenital diaphragmatic hernia should be considered.

In a similar case report, an adolescent presented with constant, nonspecific, left-sided abdominal pain. She was evaluated for a urinary tract infection, a ruptured ovarian cyst, and appendicitis. Following diagnostic laparoscopy, the patient suffered significant cardiorespiratory distress and a subsequent chest radiograph revealed a left-sided diaphragmatic hernia [8].

Left-sided late-presenting CDH often presents with acute gastrointestinal symptoms, including abdominal pain and vomiting. On the other hand, right-sided late-presenting CDH presents with chronic respiratory symptoms, including pneumonia, respiratory distress, and cough [4]. The acute onset and gastrointestinal symptom profile of left-sided CDH likely arises from rapid distension of hollow abdominal viscera into the pleural space. In contrast, the partial displacement of the liver associated with right-sided CDH leads to prolonged right lung compression, resulting in respiratory symptoms. Although late-presenting CDH can involve the right or left side, right-sided presentations are more common in younger patients: 85% of late-onset right-sided CDH diagnoses are made in children under the age of 5 years old [3]. Ultimately, when a young patient presents with respiratory and/or gastrointestinal symptoms with no apparent etiology, late-presenting CDH should be considered.

The origin of CDH is not well understood. The evidence for genetic contributions to CDH is mixed. It is estimated that 15–45% of prenatal CDH found on fetal sonography is associated with cardiac and chromosomal defects, including genetic syndromes, such as Trisomy 18 [9]. Furthermore, next-generation genome sequencing techniques have identified de novo sequence variants in 10–22% of CDH patients [2]. Nevertheless, no familial occurrence of diaphragmatic hernia has been presented in the literature [3]. Even less well-understood is the late-presenting variant of CDH. It is hypothesized that, before the herniation event, congenital diaphragmatic defects may be occluded by the spleen or liver, potentially postponing symptoms and diagnosis by imaging. As a result, these congenital defects can be missed on chest radiographs before herniation events, as seen in our patient [3–5]. In fact, in one of the largest reviews of late-presenting pediatric CDH, 14.1% of children had a normal radiograph sometime before their CDH diagnosis [3]. Moreover, not all patients with late-presenting hernias have visceral displacement in the chest, making diagnosis even more difficult to capture on imaging [3].

Ultimately, clinical presentations of late-presenting CDH are diverse. Symptoms, when present, are generally respiratory or gastrointestinal and can be concurrent. Generally, late-presenting *left-sided* herniations present with acute gastrointestinal symptoms, whereas late-presenting *right-sided* herniations present with acute or chronic respiratory symptoms. Furthermore, patients with late-presenting right-sided herniations tend to be younger than patients with late-presenting left-sided herniations [3, 4]. Late-presenting CDH is typically a low grade, isolated malformation with an uncomplicated repair process. When the correct diagnosis is made, the long-term prognosis is excellent. Pediatricians should consider this condition when evaluating patients with persistent respiratory and acute or persistent gastrointestinal symptoms.

## Data Availability

The data supporting the findings of this study are available from the corresponding author upon request.
